# Sex and interspecies differences in ESR2-expressing cell distributions in mouse and rat brains

**DOI:** 10.1186/s13293-023-00574-z

**Published:** 2023-12-18

**Authors:** Masahiro Morishita, Shimpei Higo, Kinuyo Iwata, Hirotaka Ishii

**Affiliations:** Department of Anatomy and Neurobiology, Graduate School of Medicine, Nippon Medical School, 1-1-5 Sendagi, Bunkyo-ku, Tokyo, 113-8602 Japan

**Keywords:** Mouse, Rat, Brain, Estrogen, ESR2, ERβ, Immunohistochemistry, Sexual dimorphism, Species difference

## Abstract

**Background:**

ESR2, a nuclear estrogen receptor also known as estrogen receptor β, is expressed in the brain and contributes to the actions of estrogen in various physiological phenomena. However, its expression profiles in the brain have long been debated because of difficulties in detecting ESR2-expressing cells. In the present study, we aimed to determine the distribution of ESR2 in rodent brains, as well as its sex and interspecies differences, using immunohistochemical detection with a well-validated anti-ESR2 antibody (PPZ0506).

**Methods:**

To determine the expression profiles of ESR2 protein in rodent brains, whole brain sections from mice and rats of both sexes were subjected to immunostaining for ESR2. In addition, to evaluate the effects of circulating estrogen on ESR2 expression profiles, ovariectomized female mice and rats were treated with low or high doses of estrogen, and the resulting numbers of ESR2-immunopositive cells were analyzed. Welch’s t-test was used for comparisons between two groups for sex differences, and one-way analysis of variance followed by the Tukey–Kramer test were used for comparisons among multiple groups with different estrogen treatments.

**Results:**

ESR2-immunopositive cells were observed in several subregions of mouse and rat brains, including the preoptic area, extended amygdala, hypothalamus, mesencephalon, and cerebral cortex. Their distribution profiles exhibited sex and interspecies differences. In addition, low-dose estrogen treatment in ovariectomized female mice and rats tended to increase the numbers of ESR2-immunopositive cells, whereas high-dose estrogen treatment tended to decrease these numbers.

**Conclusions:**

Immunohistochemistry using the well-validated PPZ0506 antibody revealed a more localized expression of ESR2 protein in rodent brains than has previously been reported. Furthermore, there were marked sex and interspecies differences in its distribution. Our histological analyses also revealed estrogen-dependent changes in ESR2 expression levels in female brains. These findings will be helpful for understanding the ESR2-mediated actions of estrogen in the brain.

**Supplementary Information:**

The online version contains supplementary material available at 10.1186/s13293-023-00574-z.

## Plain Language Summary

Although the brain is a major target organ of estrogens, the distribution of estrogen receptors in the brain is not fully understood. ESR2, also known as estrogen receptor β, is an estrogen receptor subtype; its localization in the brain has long been controversial because it has traditionally been difficult to detect. In the present study, we analyzed the expression sites of ESR2 in mouse and rat brains using immunohistochemistry with a well-validated antibody, PPZ0506. The immunohistochemical analysis revealed a more localized expression of ESR2 protein in brain subregions than has previously been reported. Additionally, there were clear sex and interspecies differences in the distribution of this protein. We also observed changes in ESR2 expression in the female brain in response to circulating estrogen levels. Our results, which show the precise expression profiles of ESR2 protein in rodent brains, will be helpful for understanding the ESR2-mediated actions of estrogen.

**Supplementary Information:**

The online version contains supplementary material available at 10.1186/s13293-023-00574-z.

## Highlights

- Immunohistochemical analyses using a well-validated antibody revealed that ESR2 is localized to subregions of the preoptic area, extended amygdala, hypothalamus, mesencephalon, and cerebral cortex in mouse and rat brains.
- Sex and interspecies differences in ESR2 expression were observed in some brain subregions.
- Circulating estrogen levels affected ESR2 expression in the brains of female mice and rats.

**Supplementary Information:**

The online version contains supplementary material available at 10.1186/s13293-023-00574-z.

## Background

Estrogen receptors are expressed in various brain regions and regulate both reproductive and non-reproductive processes in both sexes. Two types of nuclear receptor isoforms, ESR1 and ESR2 (also known as estrogen receptor α and β, respectively), play critical roles in estrogenic actions [1, 2]. Studies using gene knockout models and selective agonists have shown that each estrogen receptor is involved in distinct developmental and physiological events. In rodents, ESR1 is essential for sexual differentiation of the brain during development [3] as well as for the regulation of reproductive neuroendocrine function [4, 5], energy metabolism [6, 7], and social behaviors [8, 9] in adulthood; it is expressed in brain regions involved in these developmental and physiological processes. ESR2 has been implicated in several physiological processes, including reproductive neuroendocrine function [10], social behaviors [8, 11], learning and memory [12], and anxiety [13]. However, despite the importance of these phenomena, the neural mechanisms of the ESR2-mediated actions of estrogen are not well understood, largely because of the difficulties in detecting ESR2-expressing cells.

Several factors, including sex, age, and physiological conditions, can influence the actions of estrogen. Its actions presumably depend on the hormonal milieu as well as the expression levels of estrogen receptors at target sites. ESR1 is expressed in sexually dimorphic patterns in many brain regions, such as the preoptic area, bed nucleus of the stria terminalis (BNST), ventromedial hypothalamic nucleus, arcuate nucleus, and amygdala; its expression levels are markedly altered by blood estrogen levels [14, 15]. Expression of ESR2 may also be differentially regulated under various physiological conditions. In the preoptic area of female rats, for example, *ESR2* and *ESR1* mRNA levels are elevated in the diestrus and estrus phases, respectively [16]. The expression profiles of *ESR1* and *ESR2* are thus differentially regulated at mRNA levels by different hormonal environments.

Several attempts have been made to examine the distributions of ESR2-expressing cells in rodent brains using homemade and commercial antibodies; such studies have revealed that ESR1 and ESR2 exhibit different expression patterns [17–21]. For example, it has been consistently reported that ESR2 is abundantly expressed in the hypothalamic paraventricular nucleus (PVN), whereas ESR1 is absent. However, several discrepancies have been reported in ESR2 expression in other brain regions, probably because of differences in antibody specificity. Although other approaches have been attempted, such as in situ hybridization for *ERS2* mRNA [22, 23] or the generation of transgenic mice expressing fluorescent proteins under *ESR2* promoter [24–26], these techniques are unable to directly detect ESR2 protein. The lack of validated tools to detect ESR2 had led to a halt in ESR2 research. Recently, however, a report by Anderson et al. [27] has overcome this problem. These authors evaluated the specificity of commercially available antibodies against human ESR2 and identified just one anti-human ESR2 monoclonal antibody (PPZ0506) with specific immunoreactivity against human ESR2 protein. To further validate this antibody for use in ESR2 research, we confirmed its specificity and cross-reactivity against mouse and rat ESR2 and optimized its protocols for immunohistological analysis [28–30]. These studies revealed species-related differences in ESR2 expression in the peripheral tissues of mice and rats [29, 30]. However, although the brain is a primary target site of estrogen, the precise expression profiles of ESR2 in the brain remain unclear. In the present study, we aimed to identify sex and interspecies differences in ESR2 distributions in rodent brains by mapping ESR2-immunopositive (ESR2^+^) cells in the whole brains of mice and rats of both sexes. Furthermore, we examined the effects of estrogen manipulations on ESR2^+^ cell numbers in brain subregions of female mice and rats.

## Materials and methods

### Animals

We used sexually naïve 9- to 16-week-old C57BL/6J mice and Wistar rats of both sexes. The animals were housed on a 14-h light/10-h dark cycle (lights on at 8:00 a.m.) at room temperature (22 °C ± 2 °C) with free access to a standard diet and tap water.

### Experimental design

#### Mapping of ESR2^+^ cells in mice and rat brains

Gonadally intact male mice (n = 4) and rats (n = 4) were euthanized and histologically processed. Gonadally intact female mice (n = 4) and rats (n = 4) were monitored for their estrous cycles by daily vaginal smear cytology. After the confirmation of two regular estrous cycles, they were euthanized in the diestrus phase for histological processing. One series of whole brain sections from the individual animals underwent immunohistochemistry for ESR2 and Nissl staining, to examine ESR2^+^ cell distribution. In addition, ESR2^+^ cell numbers were counted in the neural nuclei in which ESR2 proteins were predominantly localized. Other series of the brain sections underwent double immunohistochemistry for ESR2/oxytocin (OXT) and ESR2/arginine vasopressin (AVP).

#### Effects of estrogen manipulation on the distribution and number of ESR2^+^ cells

**Fig. 1:**
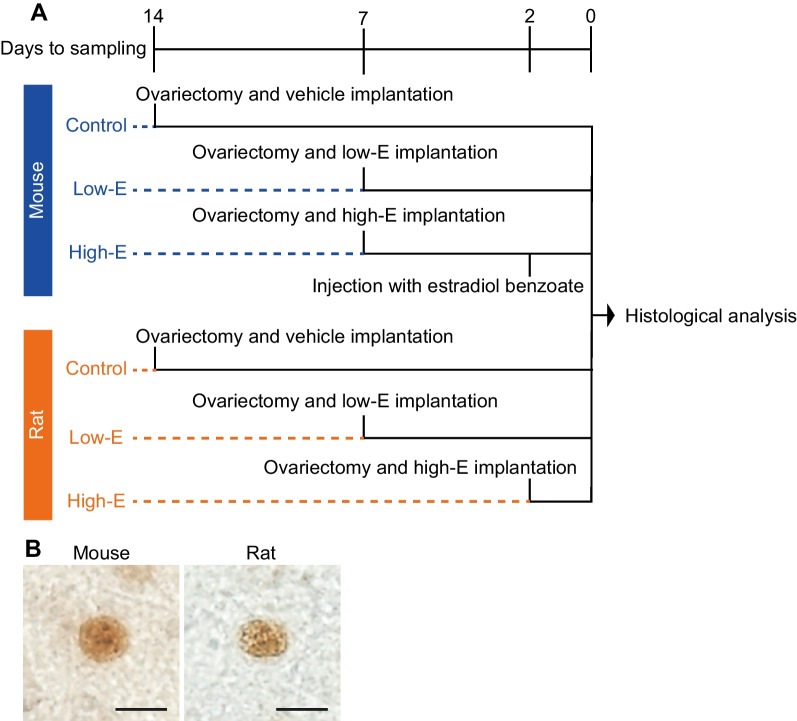
Supplement to methods. Time courses of the estrogen manipulations applied to female mice and rats (**A**). Representative photomicrographs of mouse and rat ESR2^+^ cells with immunopositive signal in the cell nuclei (**B**). Scale bars = 10 µm

After the confirmation of two regular estrous cycles by daily vaginal smear cytology, female mice (n = 15) and rats (n = 15) were randomized into three groups: ovariectomy + vehicle treatment (control; n = 5 for each species), ovariectomy + low-dose estrogen treatment (low-E; n = 5 for each species), and ovariectomy + high-dose estrogen treatment (high-E; n = 5 for each species). All female mice and rats were anesthetized by inhalation of isoflurane gas (concentration, 1%–3% in the air; flow rate, 0.5 L/min), ovariectomized, and implanted subcutaneously with a silicon tube, which was filled with different concentrations of estrogens for each experimental group (see Fig. 1A for the time-course details). For mice, the implanted silicon tube (inner diameter 1.02 mm, outer diameter 2.16 mm, length 18.0 mm, length of tube filled with substance 12.0 mm; Dow Corning Corporation, Midland, MI, USA) was filled with sesame oil for the control, 0.1 µg/µL 17β-estradiol (E_2_) in sesame oil for the low-E group, or 0.2 µg/µL E_2_ in sesame oil for the high-E group. For rats, the implanted silicon tube (inner diameter 1.57 mm, outer diameter 3.18 mm, length 37.0 mm, length of tube filled with substance 25.0 mm for the control and low-E groups; inner diameter 1.02 mm, outer diameter 2.16 mm, length 32.0 mm, length of tube filled with substance 20.0 mm for the high-E group; Dow Corning Corporation) was filled with either sesame oil for the control, 0.2 µg/µL E_2_ in sesame oil for the low-E group, or crystalline E_2_ for the high-E group. The ends of the substance-filled silicon tubes were sealed with glue (length of glue at each end: 3 mm for mice and 6 mm for rats). Six days after surgery, high-E mice were injected subcutaneously with estradiol benzoate in sesame oil (1 µg/100 µL) 3 h after light onset in the light/dark cycle. We euthanized the control mice and rats 14 days after surgery, the low-E mice and rats and high-E mice 7 days after surgery, and the high-E rats 2 days after surgery. The conditions for estrogen manipulation and brain sampling were set according to commonly used methods, with low-E treatment used to mimic estrogen levels within the physiological range that produce a negative feedback effect on luteinizing hormone pulses [31, 32], and high-E treatment leading to excessive estrogen levels that produce a positive feedback effect on luteinizing hormone surges [33, 34]. The brain sections obtained from these animals were subjected to immunostaining for ESR2 as well as Nissl staining, to examine ESR2^+^ cell numbers in the anteroventral periventricular nucleus (AVPV), medial preoptic nucleus (MPN), principal nucleus of the BNST (BNSTp), posterodorsal subnucleus of the medial amygdala (MePD), supraoptic nucleus (SON), PVN, and dorsal raphe nucleus (DRN).

### General procedures

#### Tissue preparation

Animals were deeply anesthetized by the intraperitoneal injection of mixed anesthesia: medetomidine hydrochloride (0.3 mg/kg body weight), midazolam (4.0 mg/kg body weight), and butorphanol tartrate (5.0 mg/kg body weight). The animals were then perfused transcardially with saline followed by 4% paraformaldehyde in 0.1 M phosphate buffer (pH 7.4). The brains were postfixed with the same fixative at 4 °C overnight, immersed in 0.1 M phosphate buffer containing 20% sucrose for 2–5 days at 4 °C, and then frozen in n-hexane at − 80 °C. Brains were sectioned coronally using a cryostat at a thickness of 30 µm for mice and 40 µm for rats. The mouse and rat brain sections were collected at intervals of 90 µm and 160 µm, respectively, for mapping of ESR2^+^ cells, and at intervals of 60 µm and 80 µm, respectively, for examining the effects of estrogen manipulation.

#### Characterization of the anti-ESR2 antibody

The anti-ESR2 mouse monoclonal antibody (PPZ0506, Perseus Proteomics, Tokyo, Japan; RRID:AB_1964229), raised against amino acids 2–88 from the N-terminal of human ESR2 protein, has been confirmed to specifically recognize FLAG-tagged mouse, rat, and human ESR2 proteins using western blotting and immunocytofluorescence detection [28]. In addition, our previous studies have established its applicability to the immunohistochemical detection of mouse and rat ESR2 proteins [29, 30, 35].

#### Immunohistochemistry for ESR2 and Nissl staining

Brain sections were mounted on FRONTIER-coated glass slides (Matsunami Glass, Osaka, Japan) and warmed on a slide warmer at 60 °C for 1 h. The sections were then rinsed in distilled water and placed in methanol containing 0.3% H_2_O_2_ for 5 min at room temperature. Next, they were rinsed in 0.1 M phosphate-buffered saline (PBS, pH 7.4), autoclaved at 121 °C for 10 min in citrate-based antigen unmasking solution (pH 6.0; H-3300, Vector Laboratories, Newark, CA, USA) for antigen retrieval, and rinsed again in PBS. The mouse brain sections were then blocked for 30 min at room temperature with blocking reagent (a component of the Mouse-on-Mouse Polymer IHC Kit, ab269452, Abcam, Cambridge, UK), whereas the rat brain sections were blocked for 30 min at room temperature with 5% normal goat serum in PBS containing 0.3% Triton X-100 (PBST, pH 7.4). This blocking was followed by incubation at 4 °C overnight with anti-ESR2 antibody (PPZ0506), which was dissolved in PBST (1: 2000) for the mouse brain sections and in 5% normal goat serum in PBST (1: 2000) for the rat brain sections. For the negative controls, residual brain sections from the series were exposed to solvent without the anti-ESR2 antibody. After rinsing with PBST, the mouse brain sections were incubated for 15 min at room temperature with horseradish peroxidase polymer detector reagent (a component of the Mouse-on-Mouse Polymer IHC Kit), whereas the rat brain sections were incubated for 2 h at room temperature with 50% goat anti-mouse immunoglobulin G conjugated to a horseradish peroxidase-labeled polymer (ab214879, Abcam) in PBST. All sections were then rinsed in PBS and 0.01 M Tris–HCl (pH 7.5) before the ESR2^+^ signals were visualized using 200 µg/mL of 3,3′-diaminobenzidine tetrahydrochloride (Merck, Darmstadt, Germany) and 0.01% H_2_O_2_ in 0.05 M Tris–HCl (pH 7.5). Finally, the sections were rinsed in PBS, stained with 0.025% violet solution, dehydrated through an ethanol series, cleared with xylene, and coverslipped using mounting medium (Entellan new, Merck).

#### Immunohistochemistry for OXT or AVP + ESR2

Mouse and rat brain sections were placed in PBST containing 0.3% H_2_O_2_ for 30 min at room temperature. Next, they were rinsed in 0.1 M PBS, blocked for 30 min at room temperature with 5% normal goat serum in PBST, and then immunoreacted in 5% normal goat serum in PBST containing rabbit anti-OXT antibody (1: 20,000; AB911, Millipore, MA, USA; RRID:AB_2157629) or guinea-pig anti-AVP antibody (1: 5000; T5048, Peninsula Laboratories, CA, USA; RRID:AB_518680) at 4 °C overnight. After rinsing with PBST, the brain sections were incubated for 2 h at room temperature with 50% goat anti-rabbit immunoglobulin G conjugated to a horseradish peroxidase-labeled polymer (ab214880, Abcam) in PBST or horseradish peroxidase-labeled goat anti-guinea pig immunoglobulin G (SA00001-12, Proteintech, IL, USA; RRID:AB_2890975) in PBST (1: 1000). All sections were then rinsed in PBST before the immunoreactive signals were visualized using an ImmPACT SG substrate kit (SK-4705, Vector Laboratories). After the sections were rinsed in PBST, antibodies were removed from the sections by incubation with 0.1 M of glycine–HCl buffer (pH 2.2) for 90 min at room temperature. Subsequently, OXT or AVP-immunostained sections were mounted on FRONTIER-coated glass slides, warmed on a slide warmer at 60 °C for 1 h, and subjected to ESR2 immunostaining. After ESR2 immunoreactive signals were detected, the sections were rinsed in PBS, dehydrated through an ethanol series, cleared with xylene, and coverslipped using mounting medium (Entellan new).

#### Histological analysis

Digital images of brain sections were obtained using a microscope (BX51, Olympus, Tokyo, Japan) equipped with a charge-coupled device camera (DP73, Olympus) and a microscope (DMD108, Leica Microsystems, Wetzlar, Germany); the same imaging conditions were used for the same animal species. Atlases of brains of mice and rats were used as references to identify brain regions [36, 37]. To examine sex differences and the effects of estrogen manipulation on ESR2 expression, photomicrographs of all brain sections containing the target regions were obtained after blinding the specimens. Target neural nuclei identified as clusters of Nissl-stained neurons were traced as outlines of the region of interest using ImageJ software (National Institutes of Health, Bethesda, MD, USA; RRID: SCR_003070). In the regions of interest, cell nuclei with immunopositive signals (Fig. 1B) were manually counted as ESR2^+^ cells. All paired neural nuclei were analyzed on the left side of the brain because no apparent laterality was identified. See Additional file 9: Tables S1 and Additional file 10: Table S2 for the number of brain sections used for the cell counting.

### Statistical analysis

Welch’s t-test was used to assess statistical differences between sexes. One-way analysis of variance was used to determine differences in data among females who had been ovariectomized and treated with estrogen. When significant effects were detected by one-way analysis of variance, Tukey–Kramer test was performed to compare experimental groups. Differences were considered significant at p < 0.05. GraphPad Prism software version 9.5 (GraphPad, San Diego, CA, USA; RRID:SCR_002798) was used for all statistical analyses.

## Results

### Distribution of ESR2^+^ cells in the brains of mice and rats

**Fig. 2:**
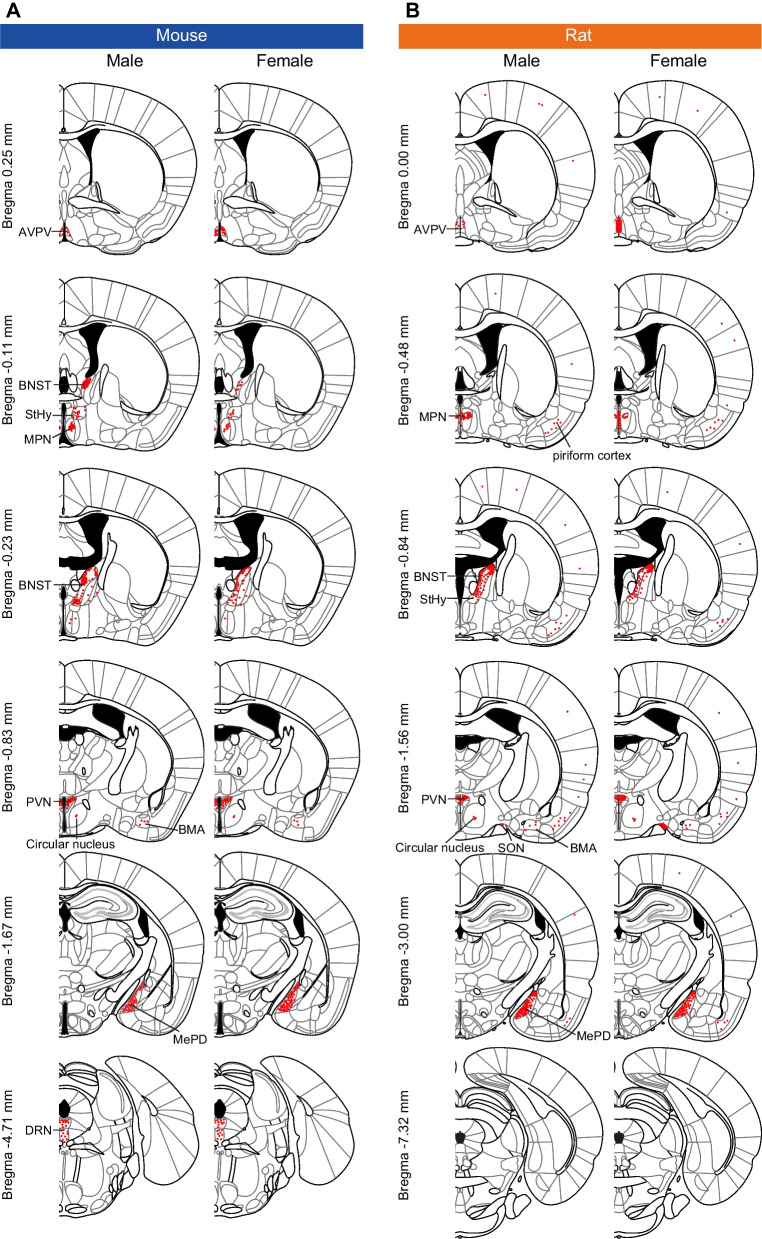
Distribution of ESR2^+^ cells in mouse and rat brains. Schematic illustrations of ESR2^+^ cell distributions in the brains of mice (**A**) and rats (**B**). Illustrations were drawn based on the Paxinos and Franklin mouse brain atlas [36] and the Paxinos and Watson rat brain atlas [37]. Red dots indicate sites where ESR2^+^ cells were commonly observed in the animals in this study

Coronal brain sections were prepared from adult gonadally intact mice and rats of both sexes before being immunostained with a well-validated anti-ESR2 antibody (PPZ0506). Overall, ESR2^+^ cells were observed in several subregions of the brain—the preoptic area, extended amygdala, hypothalamus, mesencephalon, and cerebral cortex—with similar distribution patterns between mice (Fig. 2A) and rats (Fig. 2B) of both sexes (Table 1). However, some brain subregions showed sex and/or interspecies differences in the numbers and distributions of ESR2^+^ cells. No ESR2^+^ cells were observed in the olfactory bulb, hippocampal formation, cerebellum, pons, or medulla oblongata in either mice or rats (Additional file 1: Fig. S1, Additional file 2: Fig. S2). When the primary antibody reaction was omitted, some neural fibers in the white matter were non-specifically stained, but cell nuclei were not stained (Additional file 3: Fig. S3). In particular, mouse olfactory bulbs were darkly stained regardless of the primary antibody reaction, and cell nuclei were not stained (Additional file 1: Fig. S1).

**Table 1:** Distribution of ESR2^+^ cells in the brains of mice and rats

Brain regions	Mouse		Rat	
	Male	Female	Male	Female
Telencephalon				
Pallium				
Isocortex				
Piriform cortex				
Layer 4, 5	−	−	+	+
Layer 1, 2, 3, 6	−	−	−	−
Endopiriform nu	−	−	−	−
Others		−		
Layer 4, 5	−	−	−/ +	−/+
Layer 1, 2, 3, 6	−	−	−	−
Olfactory areas				
Main olfactory bulb	−*	−*	−	−
Accessary olfactory bulb	−*	−*	−	−
Others	−	−	−	−
Hippocampal formation	−	−	−	−
Subpallium				
Claustrum	−	−	−	−
Striatum	−	−	−	−
Pallidum	−	−	−	−
Diagonal domain	−	−	−	−
Septum	−	−	−	−
Preoptic area				
Anteroventral periventricular nu	+	++	++	+++
Lateral preoptic area	−	−	−	−
Medial preoptic area	++	+	++	+
Median preoptic nu	−	−	−	−
Ventrolateral preoptic nu	−	−	−	−
Extended amygdala				
Amygdala				
Lateral amygdala nu	−	−	−	−
Basolateral amygdala nu	−	−	−	−
Basomedial amygdala nu	+	+	+	+
Posterior amygdala nu	+++	++	+++	++
Bed nu of the stria terminalis				
Lateral posterior division	++	+	++	+
Medial posterior division	+++	++	+++	++
Others	−	−	−	−
Striohypothalamic nu	+	+	+	+
Substantia innominata	−	−	−	−
Thalamus	−	−	−	−
Hypothalamus				
Periventricular hypothalamic nu	+++	+++	+++	+++
Circular nu	+	+	+	+
Supraoptic nu	−	−	−/+	++
Others	−	−	−	−
Cerebellum	−	−	−	−
Mesencephalon				
Dorsal raphe nu	++	++	−	−
Others	−	−	−	−
Rhombencephalon	−	−	−	−
Pons	−	−	−	−
Medulla	−	−	−	−

−, no ESR2^+^ cells; −/ + , no or a few ESR2^+^ cells depending on the individual; +, a few ESR2^+^ cells; ++, abundant ESR2^+^ cells; +++, very abundant ESR2^+^ cells; *, non-specific-stained; nu, nucleus

#### Preoptic area

**Fig. 3:**
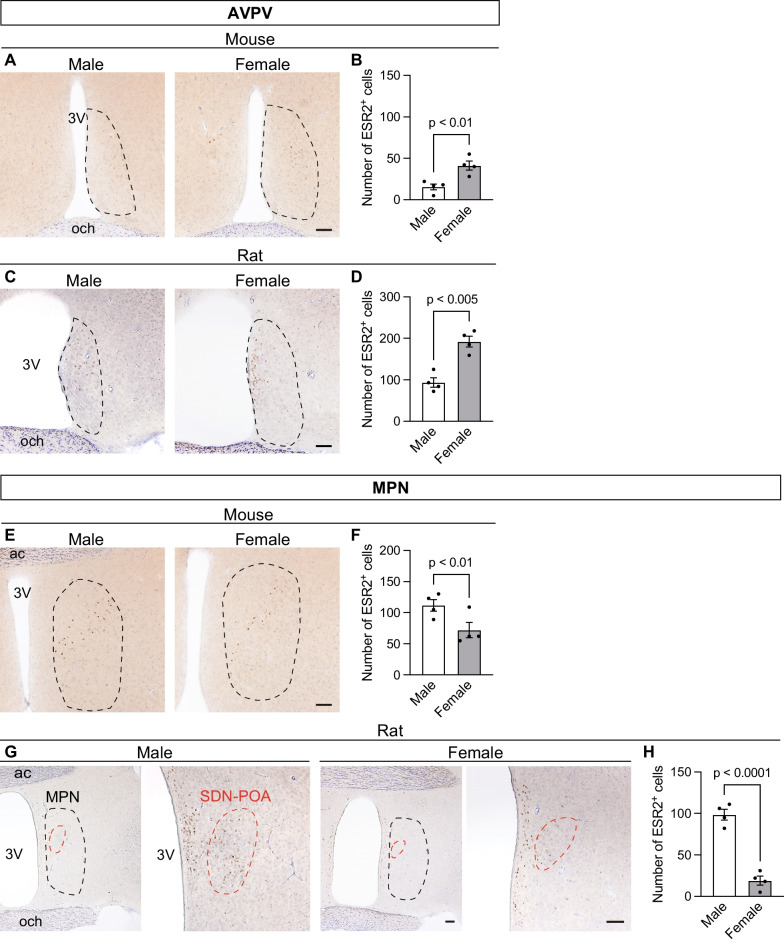
Distribution of ESR2^+^ cells in mouse and rat preoptic areas and its sex differences. Representative photomicrographs of ESR2- and Nissl-stained brain sections containing the AVPV of mice (**A**) and rats (**C**) and the MPN of mice (**E**) and rats (**G**). The black dashed line in each panel indicates the region of interest. The right images of panel G correspond to magnified views of the SDN-POA, surrounded by a red dashed line. 3 V, third ventricle; ac, anterior commissure; och, optic chiasm. Scale bars = 100 µm. The numbers of ESR2^+^ cells in the AVPV of mice (**B**) and rats (**D**) and the MPN of mice (**F**) and rats (**H**). Data are presented as the mean ± standard error of the mean (n = 4). Black dots represent individual data

In the preoptic area, ESR2^+^ cells were localized in the AVPV and MPN and sparsely distributed in their surrounding regions. There were both sex and interspecies differences in the distribution profiles of ESR2^+^ cells in the AVPV and MPN. In mice, weakly stained ESR2^+^ cells were distributed throughout the AVPV (Fig. 3A) and were more abundant in females (T_6_ = 3.88, p < 0.01; Fig. 3B). In male rats, ESR2^+^ cells were sparsely distributed in the AVPV, similar to in male mice. In contrast, in female rats, ESR2^+^ cells were restricted to the medial region of the AVPV, termed the rostral periventricular area of the third ventricle (Fig. 3C). Furthermore, ESR2^+^ cells in the rat AVPV were more abundant in females than in males (T_6_ = 5.71, p < 0.005; Fig. 3D). In both male and female rats, the ESR2^+^ cell population in the rostral periventricular area of the third ventricle of the AVPV was continuously distributed to the caudal side of the medial preoptic area. Moreover, in both the mouse and rat MPN, strongly stained ESR2^+^ cells were localized in the central region (Fig. 3E, G). In the rat MPN, ESR2^+^ cells were distributed mainly in the periphery of the Nissl-stained neuron cluster that is known as the sexually dimorphic nucleus of the preoptic area (SDN-POA). In both the mouse and rat MPN, more ESR2^+^ cells were observed in males than in females (mouse, T_6_ = 2.57, p < 0.01, Fig. 3F; rat, T_6_ = 9.46, p < 0.0001, Fig. 3H).

#### Extended amygdala

**Fig. 4:**
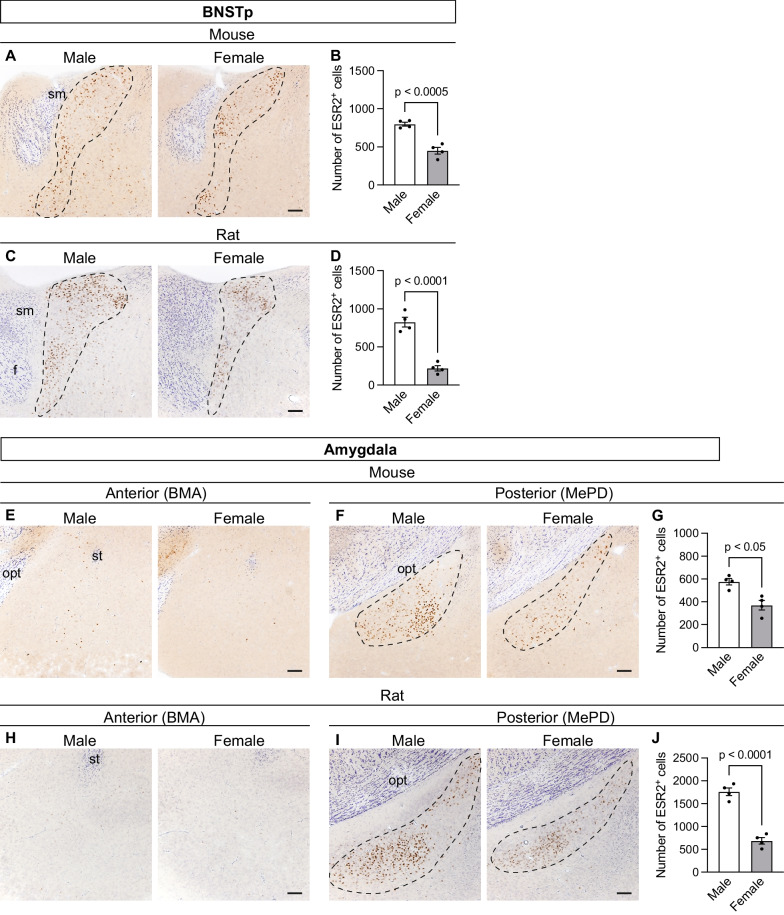
Distribution of ESR2^+^ cells in mouse and rat extended amygdalae and its sex differences. Representative photomicrographs of ESR2- and Nissl-stained brain sections containing the BNSTp of mice (**A**) and rats (**C**) and the amygdala of mice (**E** and **F**) and rats (**H** and **I**). The dashed line in each panel indicates the region of interest. f, fornix; opt, optic tract; sm, stria medullaris; st, stria terminalis. Scale bars = 100 µm. The numbers of ESR2^+^ cells in the BNSTp of mice (**B**) and rats (**D**) and the MePD of mice (**G**) and rats (**J**). Data are presented as the mean ± standard error of the mean (n = 4). Black dots represent individual data

In both mice and rats, ESR2^+^ cells were observed in several subnuclei in the extended amygdala, with no apparent interspecies differences in distribution. However, there was a male-biased difference in the number of ESR2^+^ cells in this region. In the mouse and rat BNST, ESR2^+^ cells were widely distributed in the posterior part, and were especially abundant in a subnucleus of the posterior medial region, known as the BNSTp (Fig. 4A, C). As in the SDN-POA, ESR2^+^ cells were densely distributed in the periphery of the BNSTp and sparsely distributed in the center. In addition, ESR2^+^ cells were more abundant in males than in females (mouse, T_6_ = 4.07, p < 0.0005, Fig. 4B; rat, T_6_ = 8.36, p < 0.0001, Fig. 4D). A few ESR2^+^ cells were observed in the striohypothalamic nucleus (StHy), which borders the BNST and preoptic area (Figs. 2A, B). In the amygdala, ESR2^+^ cells were diffusely distributed in and around the basomedial amygdaloid nucleus (BMA) in the anterior region (Fig. 4E, H), and were densely localized in the MePD in the posterior region (Fig. 4F, I). In the MePD, there were more ESR2^+^ cells in males than in females (mouse, T_6_ = 6.87, p < 0.05, Fig. 4G; rat, T_6_ = 8.36, p < 0.0001, Fig. 4J).

#### Hypothalamus

**Fig. 5:**
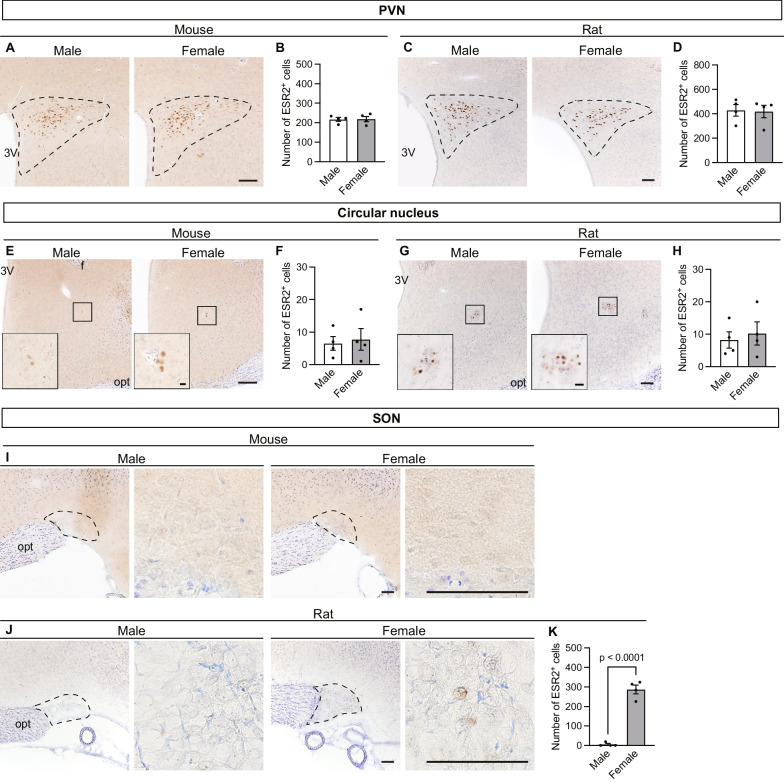
Distribution of ESR2^+^ cells in mouse and rat hypothalami and its sex differences. Representative photomicrographs of ESR2- and Nissl-stained brain sections containing the PVN of mice (**A**) and rats (**C**), the circular nucleus of mice (**E**) and rats (**G**), and the SON of mice (**I**) and rats (**J**). The dashed line in each panel indicates the region of interest. The insets of panel **E** and **G** show high-magnification views of the circular nucleus and are indicated by the small frames. 3 V, third ventricle; f, fornix; opt, optic tract. Scale bars = 10 µm in the inset of Panel **G** and 100 µm in all other images. The numbers of ESR2^+^ cells in the PVN of mice (B) and rats (D), the circular nucleus of mice (**F**) and rats (**H**), and the SON of rats (**K**). Data are presented as the mean ± standard error of the mean (n = 4). Black dots represent individual data

In both mice and rats, many ESR2^+^ cells were observed in the PVN (Fig. 5A, C). In particular, ESR2^+^ cells were densely located in the posterior and dorsolateral part of this region. There were no apparent sex or interspecies differences in ESR2^+^ cell distribution in the PVN (Fig. 5B, D). Colocalization of ESR2/OXT and ESR2/AVP was determined by double immunostaining. Most ESR2^+^ cells were OXT- and AVP-immunonegative in both mice and rats because OXT- and AVP-immunopositive cells were located mainly in the anterior part of the PVN (Additional file 4: Fig. S4, Additional file 5: Fig. S5). However, co-immunopositive cells were found in overlapping regions of the cell population. Among co-immunopositive cells, OXT-immunopositive ESR2^+^ cells were more prevalently found in mice, whereas AVP-immunopositive ESR2^+^ cells were more common in rats. In addition, a few ESR2^+^ cells were observed in the circular nucleus—a small nucleus located between the PVN and SON—in both sexes (Fig. 5E–H). In the circular nuclei, OXT-positive ESR2^+^ cells were more prevalent than AVP-positive ESR2^+^ cells. (Additional file 6: Fig. S6). In the mouse hypothalamus, ESR2^+^ cells were observed in the PVN and circular nucleus only (Fig. 5I), whereas in the rat hypothalamus, they were also observed in the SON (Fig. 5J). Although ESR2^+^ cells were densely distributed in the female rat SON of all examined, only a few ESR2^+^ cells were observed in one of the four specimens prepared from the male rat SON (no ESR2^+^ cells were detected in the other three specimens from male rats). There was thus a female-biased difference in ESR2^+^ cell number in the rat SON (T_6_ = 12.38, p < 0.0001; Fig. 5K). In the SON of female rats, more AVP-immunopositive ESR2^+^ cells were found than OXT-immunopositive ESR2^+^ cells (Additional file 7: Fig. S7).

#### Mesencephalon

**Fig. 6:**
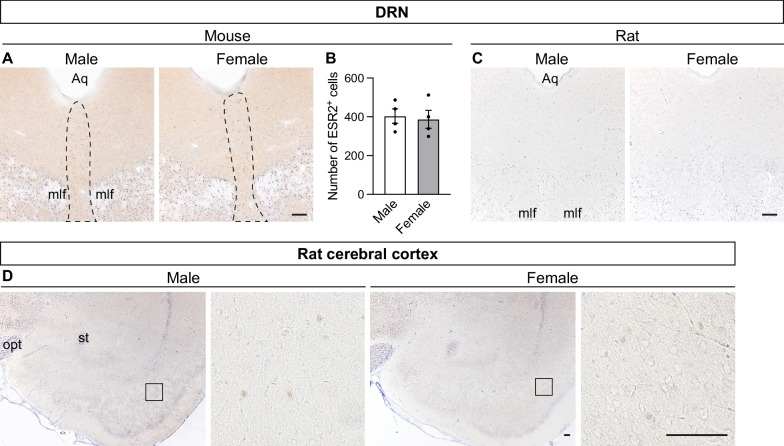
Distribution of ESR2^+^ cells in mouse and rat mesencephalons and cerebral cortexes. Representative photomicrographs of ESR2- and Nissl-stained brain sections containing the DRN of mice (**A**) and rats (**C**) and the cerebral cortex of rats (**D**). The dashed line in panel **A** indicates the region of interest. The right images of panel **D** correspond to magnified views of regions indicated by the small frames. Aq, mesencephalic aqueduct; mlf, medial longitudinal fasciculus; opt, optic tract; st, stria terminalis. Scale bars = 100 µm. The numbers of ESR2^+^ cells in the DRN of mice (**B**). Data are presented as the mean ± standard error of the mean (n = 4). Black dots represent individual data

In mice, ESR2^+^ cells were localized in the dorsal and ventral parts of the DRN (Fig. 6A), with no sex differences in distribution (Fig. 6B). In contrast, no ESR2^+^ cells were observed in any rat mesencephalic regions, including the DRN (Fig. 6C).

#### Cerebral cortex

A few weakly stained ESR2^+^ cells were diffusely distributed in the rat cerebral cortex (Fig. 2B). These ESR2^+^ cells were more abundant in the piriform cortex (5–10 cells/section) than in other areas (Fig. 6D) and were mainly localized in layers 4–5. In contrast, no ESR2^+^ cells were observed in the mouse cerebral cortex.

### Effects of estrogen manipulation on ESR2 expression in female mice and rats

Ovariectomized female mice and rats underwent estrogen manipulation before their brains were used for immunohistochemical analysis. Overall, ESR2^+^ cells were observed in estrogen-manipulated animals in the regions where ESR2 was detected in intact female mice and rats (Additional file 8: Fig. S8). The mouse SON and rat DRN did not express ESR2 even though estrogen milieu was manipulated.

**Fig. 7:**
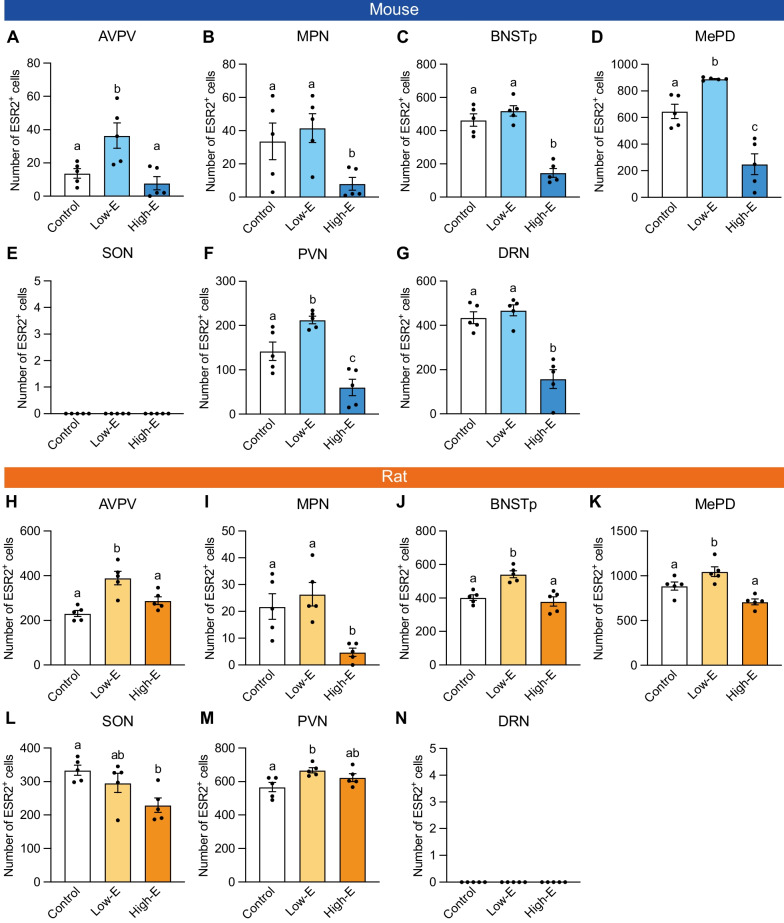
Effects of estrogen manipulation on ESR2^+^ cell number in female mouse and rat brain subregions. The number of ESR2^+^ cells in the AVPV (**A**), MPN (**B**), BNSTp (**C**), MePD (**D**), SON (**E**), PVN (**F**), and DRN (**G**) of control, low-E, and high-E female mice. The numbers of ESR2^+^ cells in the AVPV (**H**), MPN (**I**), BNSTp (**J**), MePD (**K**), SON (**L**), PVN (**M**), and DRN (**N**) of control, low-E, and high-E female rats. Data are presented as the mean ± standard error of the mean (n = 5). Black dots represent individual data. Values with different letters differ significantly (p < 0.05)

In female mice, estrogen manipulation significantly affected ESR2^+^ cell numbers in the AVPV (F_2,12_ = 8.31, p < 0.01; Fig. 7A), MPN (F_2,12_ = 4.38, p < 0.05; Fig. 7B), BNSTp (F_2,12_ = 39.71, p < 0.0001; Fig. 7C), MePD (F_2,12_ = 35.15, p < 0.0001; Fig. 7D), PVN (F_2,12_ = 20.52, p < 0.0001; Fig. 7F), and DRN (F_2,12_ = 27.78, p < 0.0001; Fig. 7G), but not in the SON (Fig. 7E). Low-E treatment significantly increased ESR2^+^ cell numbers (p < 0.05) in the AVPV, MePD, and PVN compared with controls (Fig. 7A, D, F). In contrast, high-E treatment did not affect ESR2^+^ cell number in the AVPV (Fig. 7A), but significantly decreased ESR2^+^ cell numbers (p < 0.05) in the MPN, BNSTp, MePD, PVN, and DRN (Fig. 7B–D, F, G).

In female rats, estrogen manipulation significantly affected ESR2^+^ cell numbers in the AVPV (F_2,12_ = 13.72, p < 0.001; Fig. 7H), MPN (F_2,12_ = 8.76, p < 0.005; Fig. 7I), BNSTp (F_2,12_ = 15.93, p < 0.0005; Fig. 7J), MePD (F_2,12_ = 14.08, p < 0.001; Fig. 7K), SON (F_2,12_ = 5.48, p < 0.05; Fig. 7L), and PVN (F_2,12_ = 8.31, p < 0.01; Fig. 7M), but not in the DRN (Fig. 7N). As in mice, low-E treatment significantly increased ESR2^+^ cell numbers (p < 0.05) in the AVPV, BNSTp, MePD, and PVN compared with controls (Fig. 7H, J, K, M). In contrast, high-E treatment did not affect ESR2^+^ cell numbers in the AVPV, BNSTp, MePD, or PVN (Fig. 7H, J, K, M, but significantly decreased ESR2^+^ cell numbers (p < 0.05) in the MPN and SON (Fig. 7I, L).

## Discussion

We immunostained ESR2 proteins in mouse and rat brains of both sexes using the well-validated monoclonal antibody (PPZ0506) to determine ESR2^+^ cell distributions. In this way, we revealed sex and interspecies differences in ESR2^+^ cell distributions and identified hormonal effects on their patterns in female rodent brains. The expression profiles of ESR2 in rodent brains have been a subject of great controversy, despite several attempts to study their localization. Although the details vary from report to report, previous studies using homemade or commercial antibodies have generally shown a wide distribution of ESR2 in rodent brains [17–21]. However, in situ hybridization data from the Allen brain atlas (https://mouse.brain-map.org) indicate that mouse *ESR2* mRNAs are expressed in a more localized manner in several brain regions, including the preoptic area, extended amygdala, hypothalamus, and mesencephalon. Considering the differences in results among the antibodies used, it is possible that previous reports of widespread ESR2 expression were overestimated due to false-positive antibody signals. Our immunohistochemical analysis using PPZ0506 revealed that ESR2 proteins are relatively localized in rodent brains, and our results are in good agreement with the expression patterns of *ESR2* mRNAs.

Sheng et al. [38] reported that ESR2 is expressed in the DRN of mice but not rats. In the present study, we found further species-related differences in immunoreactive signals in several brain regions: ESR2 staining was observed in the DRN of mice but not rats, and in the SON and cerebral cortex of rats but not mice. Our previous study [28] confirmed the specific cross-reactivity of PPZ0506 antibody against both mouse and rat ESR2, indicating that the distinct immunoreactive signals between mice and rats are indeed because of species-related differences in ESR2 expression. Although the neurochemical characteristics of ESR2^+^ cells in the rat cerebral cortex are unclear, it is likely that most ESR2^+^ cells in the rat SON are AVP neurons [23], whereas those in the mouse DRN are probably serotonin neurons [24]. Co-expression of ESR2 and AVP in the female rat SON is also demonstrated in the present study. These species-dependent expression profiles suggest that ESR2 may be involved in species-related differences in the effects of estrogen on the AVP and serotonin systems, although this possibility requires further investigation. In addition, although the distribution of ESR2^+^ cells in the PVN was not species-specific, species differences in neurochemical properties may be present, as indicated by the different abundance of AVP-immunopositive and OXT-immunopositive ESR2^+^ cells in mice and rats. Future research is needed to characterize the neurochemistry of the ESR2^+^ cells which were mapped in this study.

In both mice and rats, the distribution profiles of ESR2^+^ cells exhibited sex differences in several regions, most of which are known SDNs. Mouse and rat AVPVs reportedly display female-biased differences in their volumes and neuronal numbers [39, 40]. In the rat AVPV, the majority of kisspeptin neurons co-express *ESR2* mRNA [23], and there are more kisspeptin neurons in the AVPV in females than in males [41]. Furthermore, although neurons co-expressing kisspeptin and *ESR2* mRNA are scattered throughout the AVPVs of male and female mice and male rats, they are restricted to the rostral periventricular area of the third ventricle in female rats [42–44]. The species- and sex-dependent distribution profiles of ESR2^+^ cells observed in the present study are therefore in good agreement with the reports of *ESR2* mRNA-expressing cells in previous studies. Because AVPV-specific *ESR2* knockdown induces abnormal sexual cycles [42], ESR2 in the AVPV may regulate the reproductive axis via positive estrogen feedback provided by AVPV kisspeptin neurons. In contrast, ESR2^+^ cells in the MPN, BNSTp, and MePD were more abundant in males than in females in the present study. Because these SDNs reportedly have male-biased differences in their volumes and neuronal numbers [45–47], the biased distributions of ESR2^+^ cells may result from the sexually dimorphic structures of these nuclei. Moreover, in these male-biased SDNs, there are more neurons that express calbindin-D28K, androgen receptor, and/or monooxygenase DBH-like 1 in males than in females [48–51], indicating that neurons expressing these genes are candidate ERS2^+^ cells. These SDNs (i.e., the MPN, BNSTp, and MePD) are essential for the control of social and/or anxiety-like behaviors, and their functions are modulated by estrogens in both sexes [52, 53]. The sex-dependent expression profiles of ESR2 in these SDNs therefore suggest that ESR2 may modulate these phenomena. To support this idea, it has been reported that ESR2 knockdown in the medial preoptic area in the pubertal period reduces inter-male aggression, and that MePD-specific ESR2 knockdown in the adult period abolishes partner preference in male mice [54]. In addition, specific activation of BNSTp ESR2-expressing neurons leads to sexual satiety in male mice [55]. Notably, ESR2 was not expressed in the mouse SON, and the rat SON exhibited a female-biased difference in ESR2 expression. However, our previous analysis using in situ hybridization did not show any sex differences in *ESR2* mRNA expression in the rat SON [23]. Sex differences in ESR2 expression may therefore be partly caused by post-transcriptional and/or post-translational regulation.

In the present study, ESR2 expression in the brains of female mice and rats fluctuated in response to circulating estrogen levels. The effects of estrogen manipulation on ESR2^+^ cell numbers displayed similar trends in ovariectomized mice and rats: low-E treatment increased ESR2^+^ cell numbers, whereas high-E treatment decreased them. Our estrogen manipulations mimicked distinct hormonal conditions: estrogen concentrations during low-E treatment corresponded to the blood estrogen levels that cause luteinizing hormone pulses during the diestrus phase [31, 32], and those during high-E treatment corresponded to blood estrogen levels above the physiological range that causes luteinizing hormone surges [33, 34]. Our results therefore indicate that ESR2 expression in the brains of female mice and rats is maintained by estrogen at levels within the physiological range, and that estrogen depletion and exposure to excessive estrogen downregulate its expression. Our data on estrogen-dependent changes in ESR2 expression are partially consistent with other studies. Several lines of evidence indicate that high-dose estrogen treatment inhibits the expression of *ESR2* mRNA and/or protein in the AVPV, MPN, BNST, MePD, PVN, and SON [56–58]. In contrast, other studies have reported that estrogen treatment does not alter *ESR2* mRNA expression in the MPN or BNST [59], and increases its expression in the PVN [24]. The partial discrepancy between our studies and those of others is presumably caused by differences in estrogen treatment. We treated ovariectomized females with two different concentrations of estrogens by implantation for days to weeks, whereas most previous studies administered estrogens via a single subcutaneous injection. Furthermore, because ESR2 protein degradation is promoted by estradiol via the ubiquitin–proteasome system in vascular endothelial cells [60], ESR2 expression may also be regulated at the protein level in the brain, thus possibly leading to discrepancies between *ESR2* mRNA and ESR2 protein expression profiles. In the current study, low-E treatment did not affect ESR2^+^ cell numbers in the mouse MPN, BNSTp, or DRN, or in the rat MPN or SON. Because steroid hormones are synthesized de novo as neurosteroids in certain brain regions, estrogen levels in these regions are higher than circulating levels; even when circulating estrogens are undetectable after ovariectomy, local estrogen levels remain high in these regions [61]. In addition, the expression of *Cyp19a1*, an estrogen synthase, is high in the BNSTp but scarce in the AVPV in adult mice [62]. Together, these lines of evidence suggest that estrogen levels differ among neural nuclei, which may have led to the observed regional differences in the effects of estrogen manipulation.

Changes in ESR2^+^ cell numbers in response to circulating estrogen levels were insufficient to mask sexual dimorphism in the SDNs. Gonadal steroids during development have been considered essential for the sexually dimorphic formation of rodent SDNs. In the AVPV, perinatal testicular androgens are converted to estrogens in the brain, and these estrogens induce apoptotic cell death, resulting in female-biased differences in volume and neuronal number in this region [3, 63]. Neonatal estradiol benzoate treatment has been reported to masculinize the distribution patterns of *ESR2* mRNA-expressing cells in the AVPV of female rats [42]. In contrast, perinatal estrogens produce male-biased differences by inhibiting apoptotic cell death [3, 63] in the MPN and BNSTp. Peripubertal gonadal steroids are also involved in the sexually dimorphic formation of these SDNs [64, 65]. Thus, gonadal steroids during development may produce sex differences in ESR2^+^ cell numbers. Interestingly, ESR2 knockout reportedly abolishes sex differences in the number of dopamine neurons in the AVPV [40], while studies using gene knockout and selective agonist treatment have shown that ESR1, but not ESR2, is essential for the formation of sex differences in neuronal numbers in the AVPV, MPN, and BNSTp [66–68]. The contribution of ESR2 to the organizational effects of estrogens therefore remains controversial. Because perinatal estrogens upregulate ESR2 expression via ESR1 in the mouse BNSTp [69], sex differences in ESR2 expression may occur via the influence of other sex steroid receptors. However, the mechanisms underlying the formation of sex differences in ESR2 expression, and its role in the formation of SDNs, require further investigation.

Although the precise expression profiles of ESR2 obtained in the present study provide useful insights, they are limited by the sensitivity of immunohistochemistry. We have previously reported that the immunoreactive signals obtained using our methods are weaker than those obtained using high-sensitivity in situ hybridization [30]. For example, immunoreactive signals were not detectable in some brain regions where *ESR2* mRNA expression has been reported, such as the hippocampus, lateral septum, median preoptic area, and arcuate nucleus [23, 24]. It therefore cannot be excluded that ESR2 proteins may be present below the detection limit in regions without immunopositive signals. Future work will need to verify the expression of ESR2 in such regions using more sensitive methods. In addition, the high-E dosing protocol for mice commonly used to induce an LH surge followed that of Bronson et al. [34], which requires an additional subcutaneous injection of estradiol benzoate in the high-E group compared to the other treatment groups. Since vehicle injections were not performed in the other treatment groups, it cannot be excluded that this difference in the protocols may have influenced the results.

## Perspectives and significance

Immunohistochemical detection using the reliable antibody PPZ0506 revealed the distributions of ESR2^+^ cells in mouse and rat brains of both sexes. Our analyses demonstrated the presence of sex and interspecies differences in ESR2^+^ cell distributions, suggesting that caution should be exercised when extrapolating ESR2 data across species and sexes. Furthermore, our results indicate that ESR2^+^ cell numbers in female rodent brains fluctuate in response to circulating estrogen levels. Because the precise expression profiles of ESR2 proteins in rodent brains had not been previously described, our findings will be helpful for understanding the ESR2-mediated actions of estrogen in the brain.

### Supplementary Information

**Additional file 1: Figure S1.** ESR2-immunonegative regions of mouse brain. Representative photomicrographs of sections of the olfactory bulb, hippocampus, cerebellum, pons, and medulla oblongata of mice. The brain sections of male and female mice were ESR2-immunostained and Nissl-stained. Negative control sections were prepared by omitting the primary antibody. The lower images correspond to magnified views of regions indicated by the small frames. Scale bars = 1 mm in the upper panels and 100 µm in the lower panels.

**Additional file 2: Figure S2.** ESR2-immunonegative regions of rat brain. Representative photomicrographs of sections of the olfactory bulb, hippocampus, cerebellum, pons, and medulla oblongata of rats. The brain sections of male and female rats were ESR2-immunostained and Nissl-stained. Negative control sections were prepared by omitting the primary antibody. Scale bars = 1 mm in the upper panels and 100 µm in the lower panels.

**Additional file 3: Figure S3.** Representative photomicrographs of mouse and rat brain tissues without primary antibody during the staining process. Dashed lines indicate the BNSTp, identified as clusters of Nissl-stained neurons. f, fornix; sm, stria medullaris. Scale bars = 100 µm.

**Additional file 4: Figure S4.** Cellular co-localization of ESR2 and OXT or AVP in the mouse PVN. Representative photomicrographs of ESR2- (*brown)* and OXT- (*blue-gray*) immunostained brain sections containing the PVN of males (A) and females (B). Representative photomicrographs of ESR2- (*brown)* and AVP- (*blue-gray*) immunostained brain sections containing the PVN of males (C) and females (D). The lower images correspond to magnified views of regions indicated by the small frames. Scale bars = 100 µm in the upper panels and 50 µm in the lower panels.

**Additional file 5: Figure S5.** Cellular co-localization of ESR2 and OXT or AVP in the rat PVN. Representative photomicrographs of ESR2- (*brown)* and OXT- (*blue-gray*) immunostained brain sections containing the PVN of males (A) and females (B). Representative photomicrographs of ESR2- (*brown)* and AVP- (*blue-gray*) immunostained brain sections containing the PVN of males (C) and females (D). The lower images correspond to magnified views of regions indicated by the small frames. Scale bars = 100 µm in the upper panels and 50 µm in the lower panels.

**Additional file 6: Figure S6.** Cellular co-localization of ESR2 and OXT or AVP in the circular nucleus of mice and rats. Representative photomicrographs of ESR2- (*brown)* and OXT- (*blue-gray*) immunostained brain sections containing the circular nucleus of mice (A) and rats (C). Representative photomicrographs of ESR2- (*brown)* and AVP- (*blue-gray*) immunostained brain sections containing the circular nucleus of mice (B) and rats (D). Scale bars = 50 µm.

**Additional file 7: Figure S7.** Cellular co-localization of ESR2 and OXT or AVP in the female rat SON. Representative photomicrographs of ESR2- (*brown)* and OXT- (*blue-gray*) immunostained brain sections containing the SON of female rats (A). Representative photomicrographs of ESR2- (*brown)* and AVP- (*blue-gray*) immunostained brain sections containing the SON of female rats (B). The right images correspond to magnified views of regions indicated by the small frames. Scale bars = 100 µm in the left panels and 50 µm in the right panels.

**Additional file 8: Figure S8.** Representative photomicrographs of immunostained brain sections of estrogen-manipulated female mice and rats. Representative photomicrographs of ESR2- and Nissl-stained brain sections containing the AVPV, MPN, BNSTp, MePD, SON, PVN, and DRN of female mice and rats with control, low-E, and high-E. Scale bars = 100 µm.

**Additional file 9: Table S1.** The number of brain sections of intact mice and rats used for ESR2^+^ cell counting.

**Additional file 10: Table S2.** The number of brain sections of estrogen-manipulated mice and rats used for ESR2^+^ cell counting.

## Data Availability

The datasets analyzed during the current study are available from the corresponding author on reasonable request.
