# Supplementary figures and images for: Sex and interspecies differences in ESR2-expressing cell distributions in mouse and rat brains
Source: Biol Sex Differ. 2023 Dec 18;14:89. doi: 10.1186/s13293-023-00574-z (PMC10726529; doi:10.1186/s13293-023-00574-z)

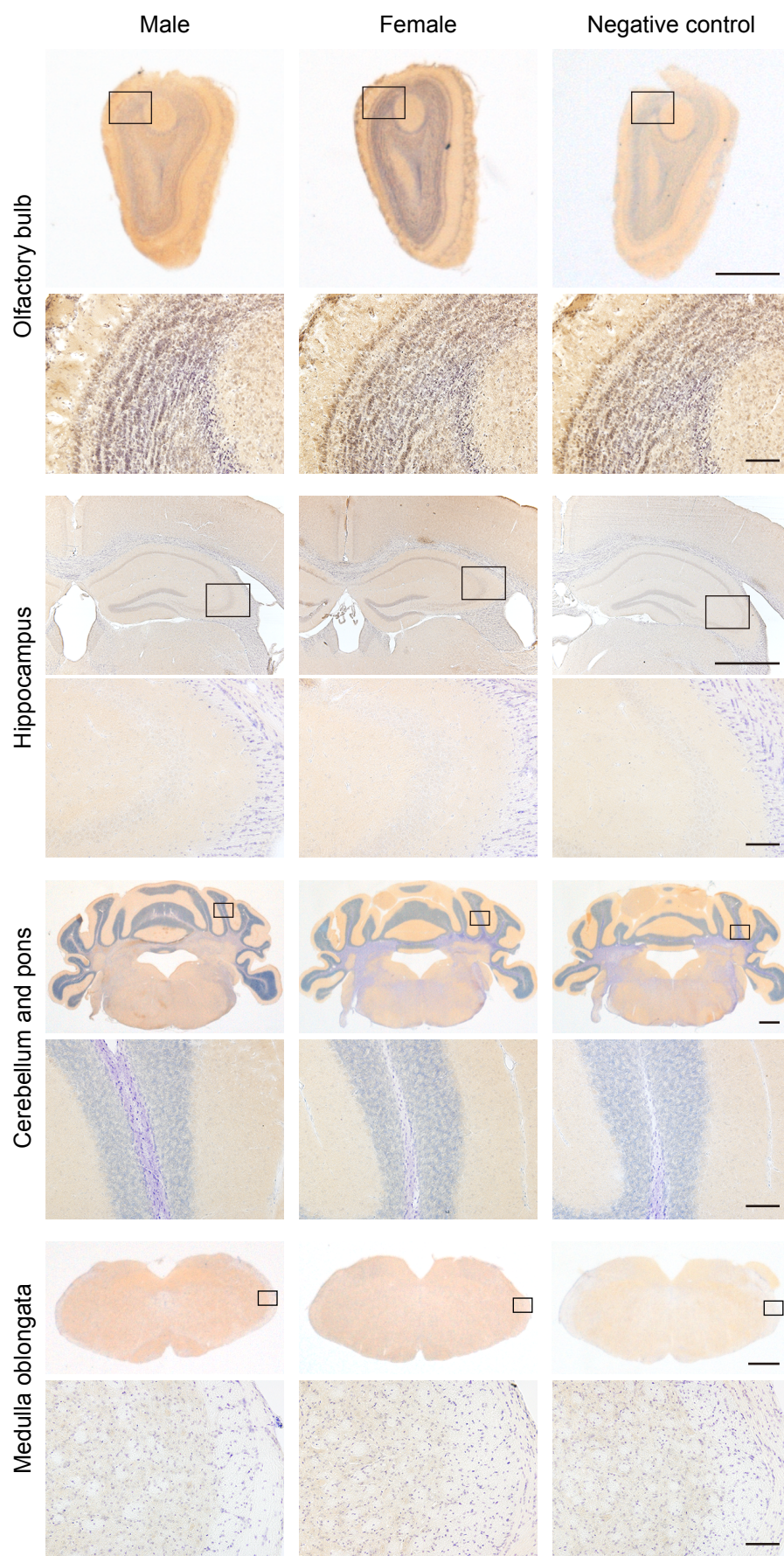

Supplement: Supplementary file 1 — Additional file 1: Figure S1. ESR2-immunonegative regions of mouse brain. Representative photomicrographs of sections of the olfactory bulb, hippocampus, cerebellum, pons, and medulla oblongata of mice. The brain sections of male and female mice were ESR2-immunostained and Nissl-stained. Negative control sections were prepared by omitting the primary antibody. The lower images correspond to magnified views of regions indicated by the small frames. Scale bars = 1 mm in the upper panels and 100 µm in the lower panels. [file 13293_2023_574_MOESM1_ESM.pdf]

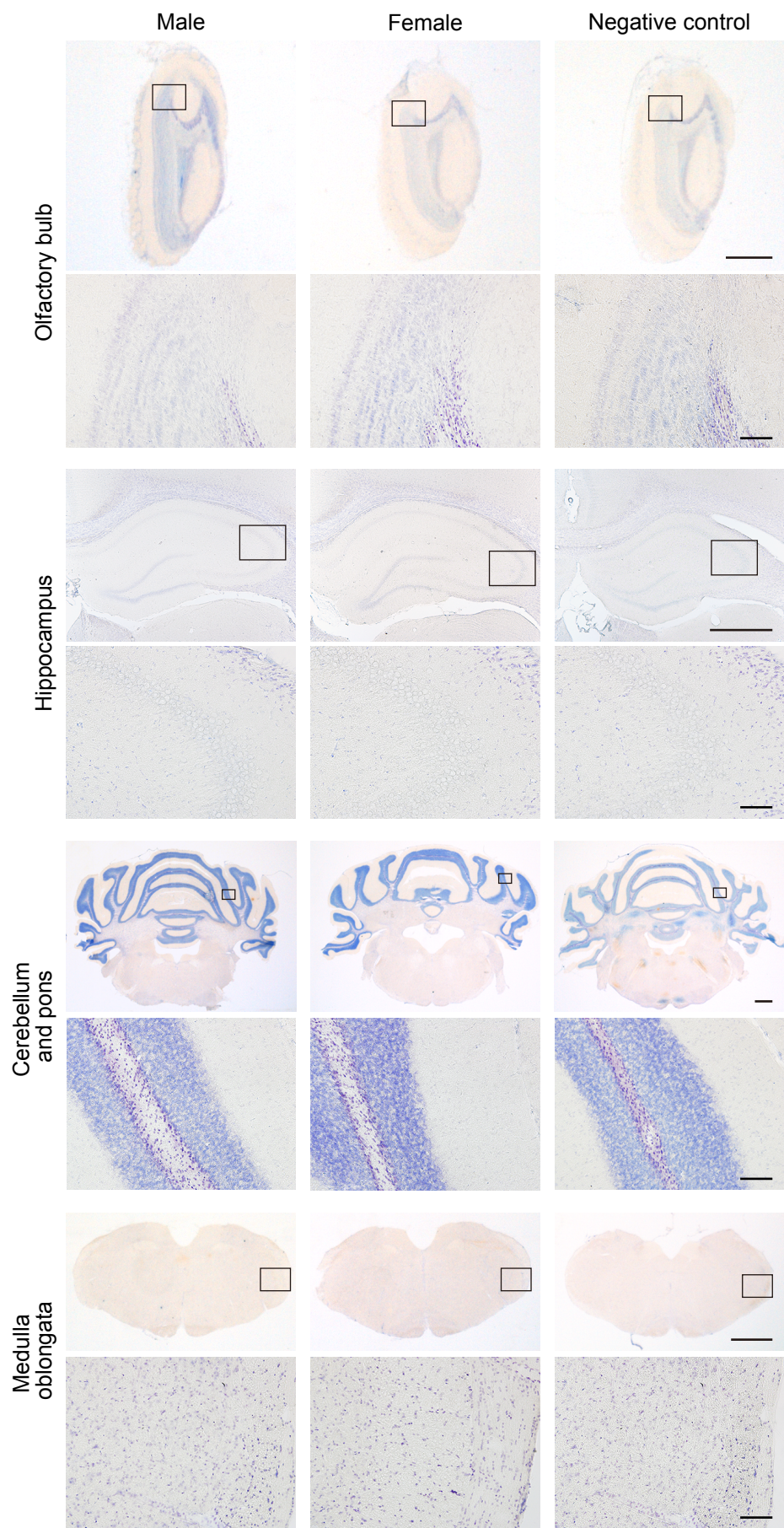

Supplement: Supplementary file 2 — Additional file 2: Figure S2. ESR2-immunonegative regions of rat brain. Representative photomicrographs of sections of the olfactory bulb, hippocampus, cerebellum, pons, and medulla oblongata of rats. The brain sections of male and female rats were ESR2-immunostained and Nissl-stained. Negative control sections were prepared by omitting the primary antibody. Scale bars = 1 mm in the upper panels and 100 µm in the lower panels. [file 13293_2023_574_MOESM2_ESM.pdf]

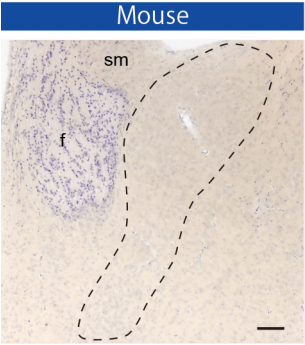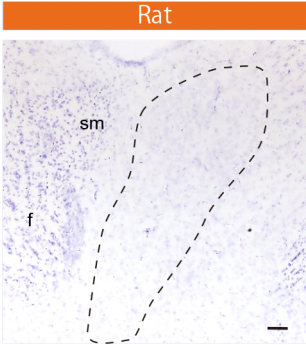

Supplement: Supplementary file 3 — Additional file 3: Figure S3. Representative photomicrographs of mouse and rat brain tissues without primary antibody during the staining process. Dashed lines indicate the BNSTp, identified as clusters of Nissl-stained neurons. f, fornix; sm, stria medullaris. Scale bars = 100 µm. [file 13293_2023_574_MOESM3_ESM.pdf]

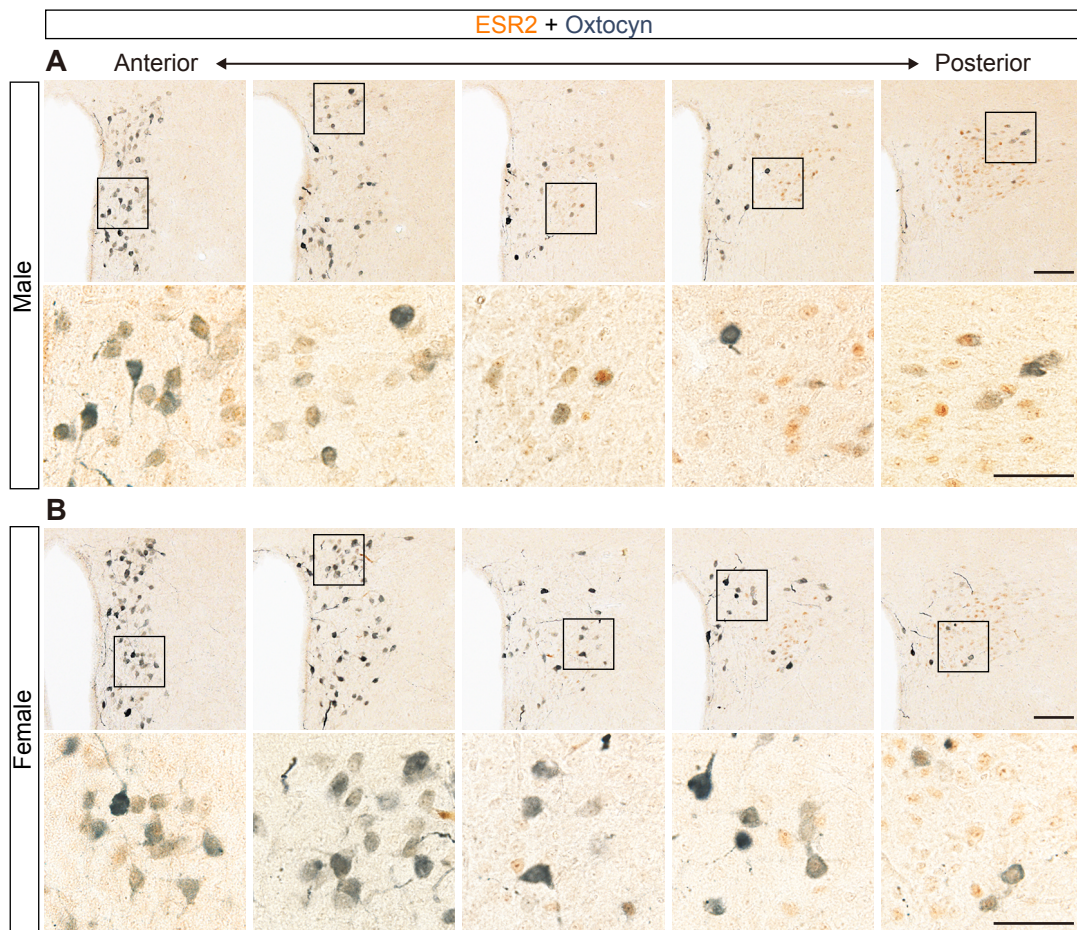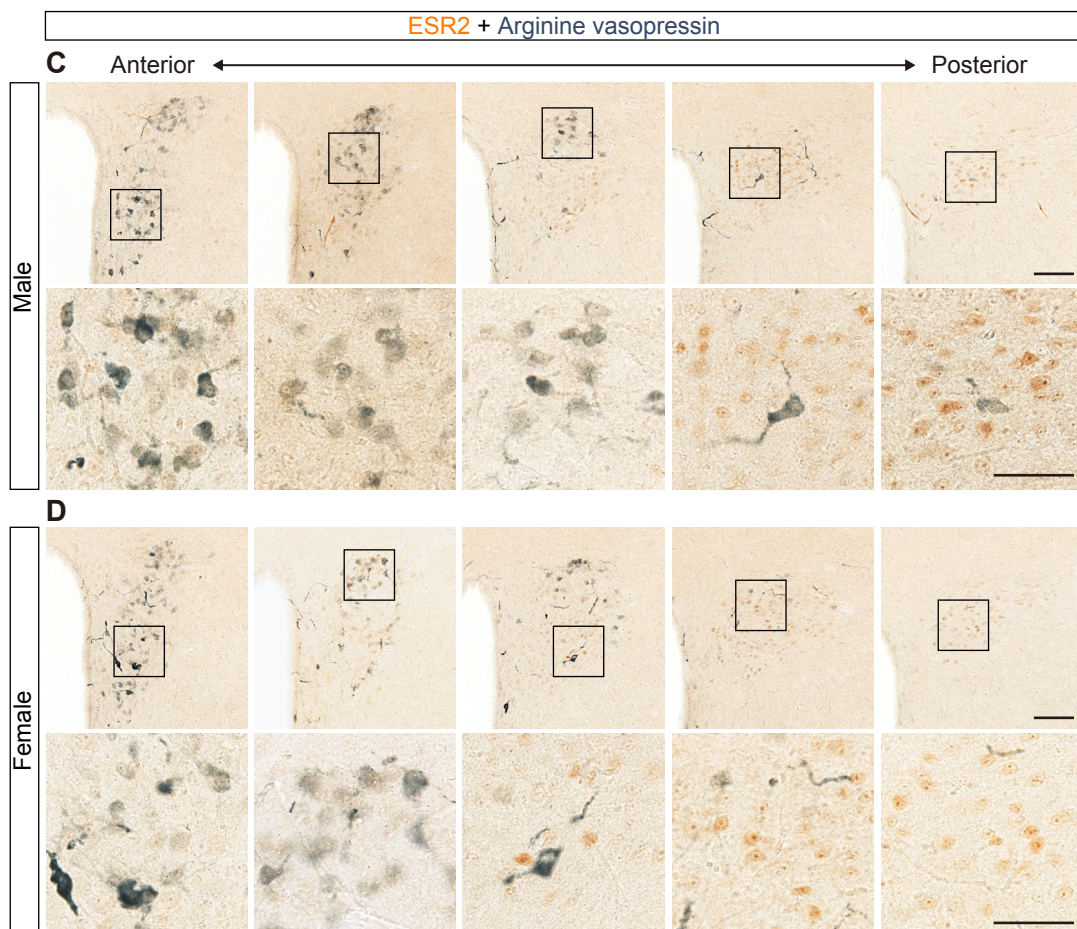

Supplement: Supplementary file 4 — Additional file 4: Figure S4. Cellular co-localization of ESR2 and OXT or AVP in the mouse PVN. Representative photomicrographs of ESR2- (brown) and OXT- (blue-gray) immunostained brain sections containing the PVN of males (A) and females (B). Representative photomicrographs of ESR2- (brown) and AVP- (blue-gray) immunostained brain sections containing the PVN of males (C) and females (D). The lower images correspond to magnified views of regions indicated by the small frames. Scale bars = 100 µm in the upper panels and 50 µm in the lower panels. [file 13293_2023_574_MOESM4_ESM.pdf]

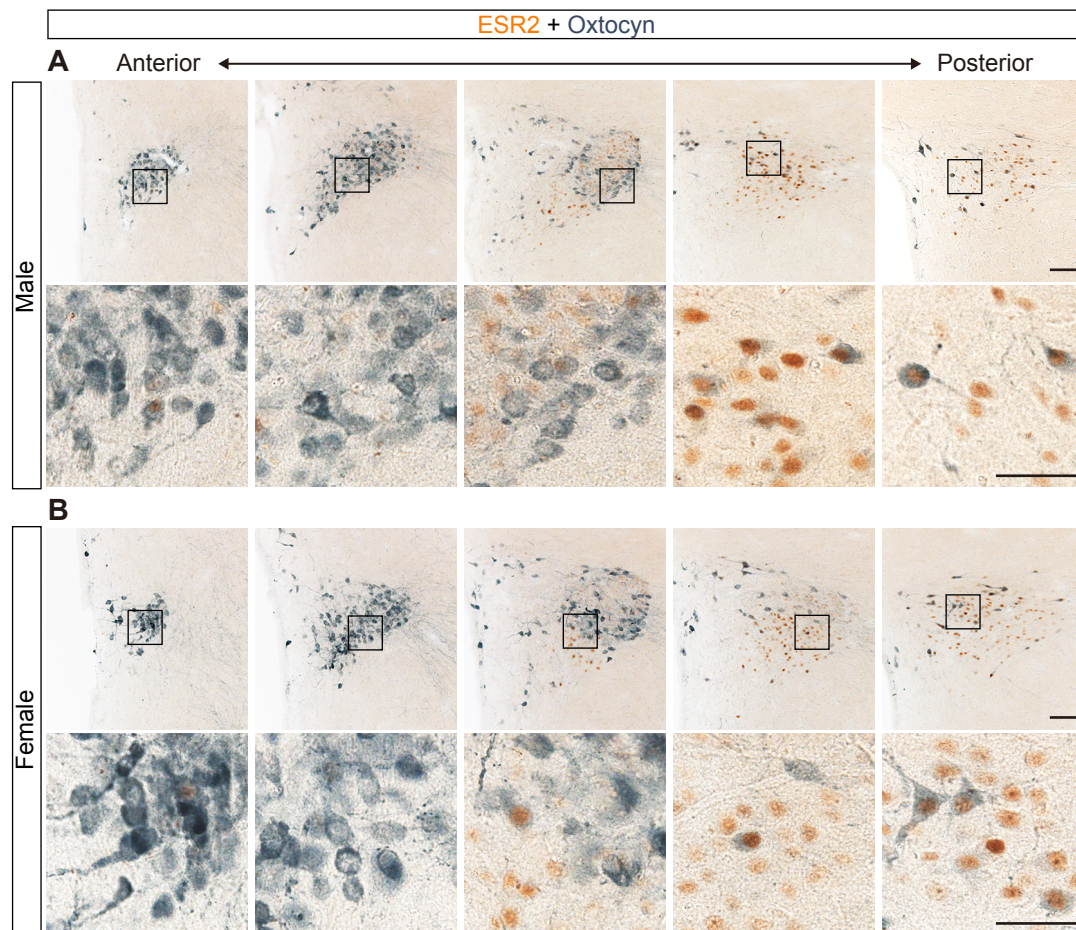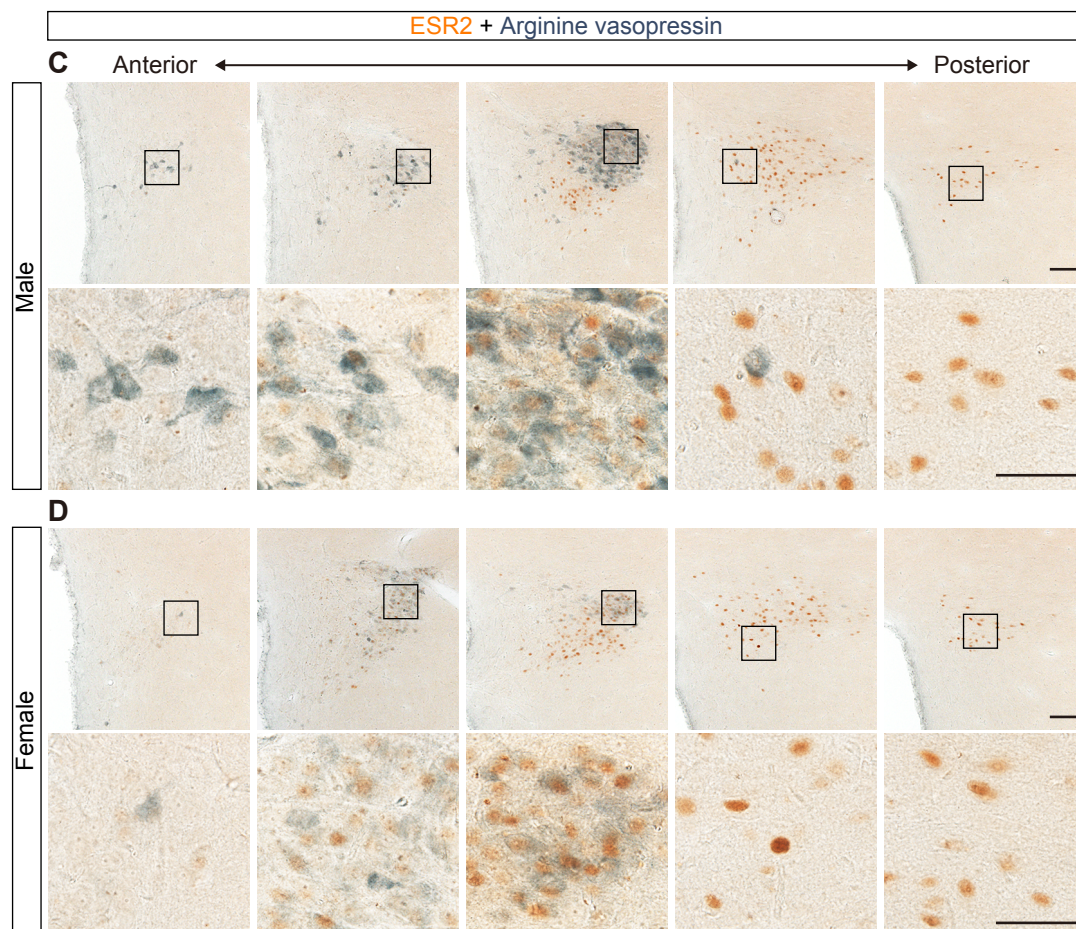

Supplement: Supplementary file 5 — Additional file 5: Figure S5. Cellular co-localization of ESR2 and OXT or AVP in the rat PVN. Representative photomicrographs of ESR2- (brown) and OXT- (blue-gray) immunostained brain sections containing the PVN of males (A) and females (B). Representative photomicrographs of ESR2- (brown) and AVP- (blue-gray) immunostained brain sections containing the PVN of males (C) and females (D). The lower images correspond to magnified views of regions indicated by the small frames. Scale bars = 100 µm in the upper panels and 50 µm in the lower panels. [file 13293_2023_574_MOESM5_ESM.pdf]

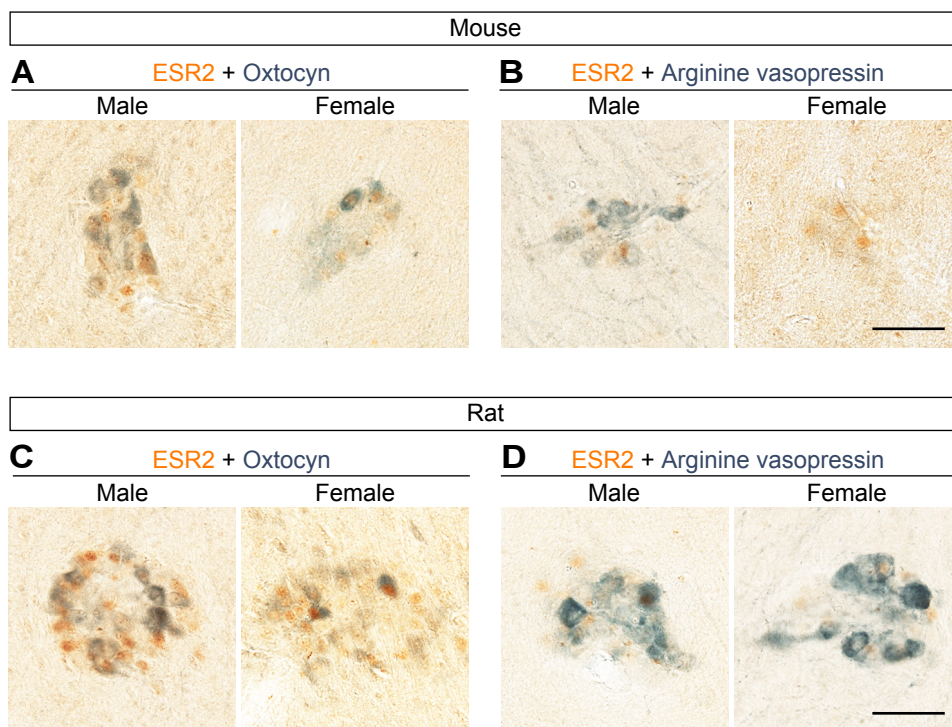

Supplement: Supplementary file 6 — Additional file 6: Figure S6. Cellular co-localization of ESR2 and OXT or AVP in the circular nucleus of mice and rats. Representative photomicrographs of ESR2- (brown) and OXT- (blue-gray) immunostained brain sections containing the circular nucleus of mice (A) and rats (C). Representative photomicrographs of ESR2- (brown) and AVP- (blue-gray) immunostained brain sections containing the circular nucleus of mice (B) and rats (D). Scale bars = 50 µm. [file 13293_2023_574_MOESM6_ESM.pdf]

**A**

ESR2 + Oxtocyn

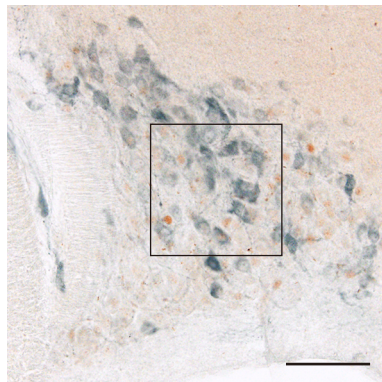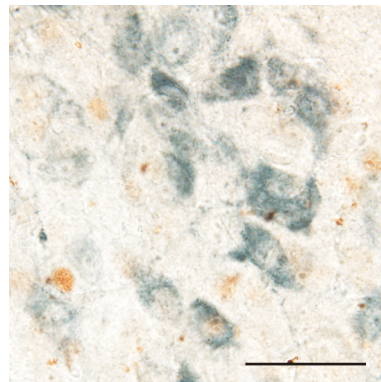

**B**

ESR2 + Arginine vasopressin

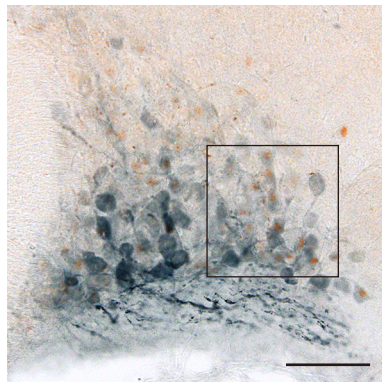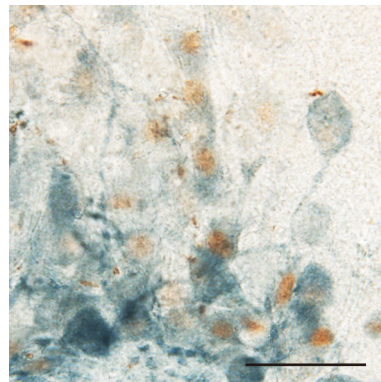

Supplement: Supplementary file 7 — Additional file 7: Figure S7. Cellular co-localization of ESR2 and OXT or AVP in the female rat SON. Representative photomicrographs of ESR2- (brown) and OXT- (blue-gray) immunostained brain sections containing the SON of female rats (A). Representative photomicrographs of ESR2- (brown) and AVP- (blue-gray) immunostained brain sections containing the SON of female rats (B). The right images correspond to magnified views of regions indicated by the small frames. Scale bars = 100 µm in the left panels and 50 µm in the right panels. [file 13293_2023_574_MOESM7_ESM.pdf]

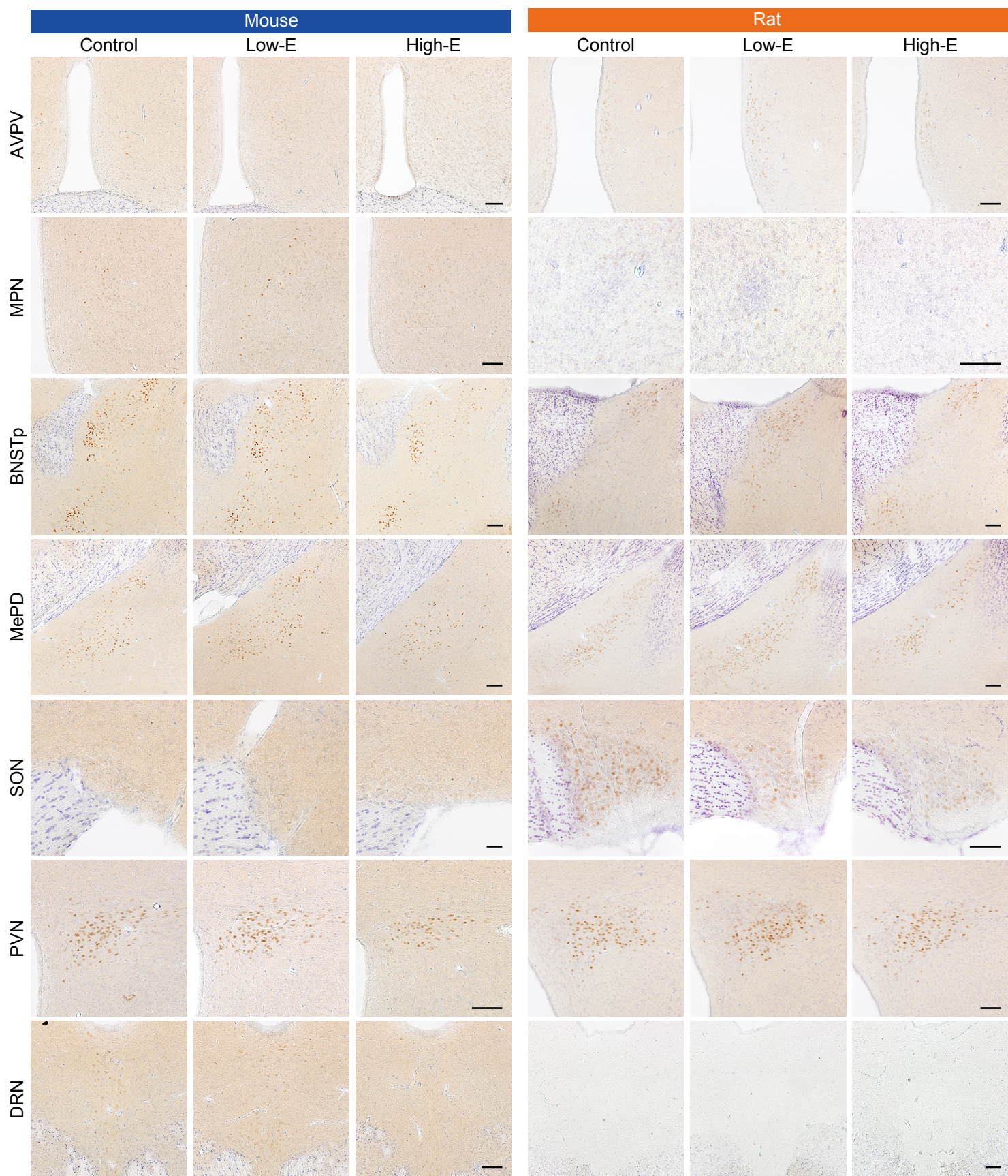

Supplement: Supplementary file 8 — Additional file 8: Figure S8. Representative photomicrographs of immunostained brain sections of estrogen-manipulated female mice and rats. Representative photomicrographs of ESR2- and Nissl-stained brain sections containing the AVPV, MPN, BNSTp, MePD, SON, PVN, and DRN of female mice and rats with control, low-E, and high-E. Scale bars = 100 µm. [file 13293_2023_574_MOESM8_ESM.pdf]
