# Supplementary material for: Sex and interspecies differences in ESR2-expressing cell distributions in mouse and rat brains
Source: Biol Sex Differ. 2023 Dec 18;14:89. doi: 10.1186/s13293-023-00574-z (PMC10726529; doi:10.1186/s13293-023-00574-z)
Supplement: Supplementary file 9 — Additional file 9: Table S1. The number of brain sections of intact mice and rats used for ESR2+ cell counting. [file 13293_2023_574_MOESM9_ESM.docx]

Supplemental table 1. The number of brain sections of intact mice and rats used for ESR2^+^ cell counting.

|  | Mouse | | Rat | |
| --- | --- | --- | --- | --- |
|  | Male | Female | Male | Female |
| AVPV | 3.25 ± 0.25 | 4.00 ± 0.41 | 3.00 ± 0.00 | 4.00 ± 0.41 |
| MPN | 3.75 ± 0.25 | 3.75 ± 0.48 | 4.50 ± 0.29 | 4.25 ± 0.25 |
| BNSTp | 4.75 ± 0.25 | 4.00 ± 0.00 | 4.50 ± 0.29 | 4.00 ± 0.41 |
| MePD | 8.75 ± 0.48 | 8.25 ± 0.25 | 7.25 ± 0.25 | 7.25 ± 0.25 |
| PVN | 7.00 ± 0.41 | 7.00 ± 0.00 | 6.25 ± 0.25 | 6.505 ± 0.29 |
| Circular nucleus | 3.00 ± 0.41 | 3.00 ± 0.00 | 2.75 ± 0.25 | 3.00 ± 0.00 |
| SON | - | - | 8.00 ± 0.00 | 7.50 ± 0.50 |
| DRN | 9.25 ± 0.63 | 9.25 ± 0.25 | - | - |

Data are presented as the mean ± standard error of the mean (n = 4).
