# Supplementary material for: Sex and interspecies differences in ESR2-expressing cell distributions in mouse and rat brains
Source: Biol Sex Differ. 2023 Dec 18;14:89. doi: 10.1186/s13293-023-00574-z (PMC10726529; doi:10.1186/s13293-023-00574-z)
Supplement: Supplementary file 10 — Additional file 10: Table S2. The number of brain sections of estrogen-manipulated mice and rats used for ESR2+ cell counting. [file 13293_2023_574_MOESM10_ESM.docx]

Supplemental table 2. The number of brain sections of estrogen-manipulated mice and rats used for ESR2^+^ cell counting.

|  | Mouse | | |
| --- | --- | --- | --- |
|  | Control | Low-E | High-E |
| AVPV | 4.60 ± 0.24 | 4.60 ± 0.24 | 4.40 ± 0.24 |
| MPN | 5.20 ± 0.37 | 5.20 ± 0.49 | 5.00 ± 0.45 |
| BNSTp | 6.00 ± 0.45 | 6.20 ± 0.73 | 6.00 ± 0.45 |
| MePD | 11.00 ± 0.45 | 10.80 ± 0.49 | 11.20 ± 0.73 |
| SON | 10.20 ± 0.20 | 10.80 ± 0.37 | 11.40 ± 0.45 |
| PVN | 10.60 ± 0.51 | 10.40 ± 0.51 | 10.60 ± 0.51 |
| DRN | 11.80 ± 0.58 | 11.60 ± 0.75 | 11.00 ± 0.71 |
|  | Rat | | |
|  | Control | Low-E | High-E |
| AVPV | 7.40 ± 0.40 | 7.60 ± 0.24 | 7.80 ± 0.20 |
| MPN | 7.80 ± 0.49 | 7.80 ± 0.49 | 7.40 ± 0.60 |
| BNSTp | 7.40 ± 0.60 | 7.00 ± 0.32 | 7.40 ± 0.40 |
| MePD | 13.00 ± 0.45 | 12.80 ± 0.73 | 12.60 ± 0.24 |
| SON | 15.40 ± 0.40 | 16.00 ± 0.00 | 15.40 ± 1.03 |
| PVN | 12.80 ± 0.73 | 12.40 ± 0.68 | 12.00 ± 0.20 |
| DRN | 19.40 ± 0.40 | 18.60 ± 0.40 | 19.40 ± 0.40 |

Data are presented as the mean ± standard error of the mean (n = 5).
